# Smart Titanium Wire Used for the Evaluation of Hydrophobic/Hydrophilic Interaction by In-Tube Solid Phase Microextraction

**DOI:** 10.3390/molecules27072353

**Published:** 2022-04-06

**Authors:** Yuping Zhang, Ning Wang, Zhenyu Lu, Na Chen, Chengxing Cui, Xinxin Chen

**Affiliations:** 1 College of Chemistry and Materials Engineering, Hunan University of Arts and Science, Changde 415000, China; 2 College of Chemistry and Chemical Engineering, Henan Institute of Science and Technology, Xinxiang 453000, China; wangning911420@163.com (N.W.); luzy_1988@163.com (Z.L.); woniunana22@163.com (N.C.); chengxingcui@hist.edu.cn (C.C.); chenxinxin920@163.com (X.C.)

**Keywords:** titanium wire, superhydrophobic, superhydrophilic, in-tube solid-phase microextraction, high-performance liquid chromatography

## Abstract

Evaluation of the hydrophobic/hydrophilic interaction individually between the sorbent and target compounds in sample pretreatment is a big challenge. Herein, a smart titanium substrate with switchable surface wettability was fabricated and selected as the sorbent for the solution. The titanium wires and meshes were fabricated by simple hydrothermal etching and chemical modification so as to construct the superhydrophilic and superhydrophobic surfaces. The micro/nano hierarchical structures of the formed TiO_2_ nanoparticles in situ on the surface of Ti substrates exhibited the switchable surface wettability. After UV irradiation for about 15.5 h, the superhydrophobic substrates became superhydrophilic. The morphologies and element composition of the wires were observed by SEM, EDS, and XRD, and their surface wettabilities were measured using the Ti mesh by contact angle goniometer. The pristine hydrophilic wire, the resulting superhydrophilic wire, superhydrophobic wire, and the UV-irradiated superhydrophilic wire were filled into a stainless tube as the sorbent instead of the sample loop of a six-port valve for on-line in-tube solid-phase microextraction. When employed in conjunction with HPLC, four kinds of wires were comparatively applied to extract six estrogens in water samples. The optimal conditions for the preconcentration and separation of target compounds were obtained with a sample volume of 60 mL, an injection rate of 2 mL/min, a desorption time of 2 min, and a mobile phase of acetonile/water (47/53, *v/v*). The results showed that both the superhydrophilic wire and UV-irradiated wire had the highest extraction efficiency for the polar compounds of estrogens with the enrichment factors in the range of 20–177, while the superhydrophobic wire exhibited the highest extraction efficiency for the non-polar compounds of five polycyclic aromatic hydrocarbons (PAHs). They demonstrated that extraction efficiency was mainly dependent on the surface wettability of the sorbent and the polarity of the target compounds, which was in accordance with the molecular theory of like dissolves like.

## 1. Introduction

As an efficient sample preparation technique, solid-phase microextraction (SPME) can integrate sampling, preconcentration, extraction, and sample injection into one step [1,2]. At present, it has been widely applied in the field of environmental analysis, drug monitoring, food testing, and biological analysis in order to remove impurities and enrich the trace target compounds in real samples [3,4,5,6]. It uses the adsorption of a sorbent to extract analytes from a sample matrix; these analytes are then desorbed from the sorbent and directed into an analytical instrument, such as gas chromatography (GC), high-performance liquid chromatography (HPLC), etc. [7,8,9,10]. The material used in the extraction coating is an important factor because the extraction process is achieved through a distribution equilibrium between the target compounds in the sample solution and the extraction coating.

At present, most commercial SPME fibers are made of fused silica, which is not only expensive, but also easy to break and swell in organic solvents [11,12]. Therefore, it is urgent to find a kind of fiber with thermal, chemical, and mechanical stability, excellent selectivity, and high sensitivity to overcome these problems. In the past two decades, many studies have focused on high-strength metal substrates, such as aluminum wire [13], silver wire [14], zinc wire [15], platinum wire [16], copper wire [17], and stainless steel wire [18], that were modified with different kinds of organic, inorganic, and hybrid coatings, which exhibited good bending properties and chemical and mechanical stabilities. Titanium dioxide (TiO_2_) has been comprehensively studied and used in various fields due to its chemical and thermal stability, biocompatibility, anti-polluting nature, and high corrosion resistance due to its photoelectrochemical activities [19]. Some studies have found that nanostructured TiO_2_ is an excellent adsorbent for organic compounds in SPE and SPME [20,21]. Moreover, it is an import smart material for many practical applications, including self-cleaning, solar cells, lithium-ion batteries, pollutant photodegradation, and oil/water separation [22,23,24,25]. In situ fabrication of a TiO_2_-nanotube coating on the surface of chemically oxidized Ti wire with hydrogen peroxide solution has been used for SPME of dichlorodiphenyltrichloroethane and its degradation products [26]. Liu et al. fabricated TiO_2_ nanotube arrays on the surface of Ti wire for use in the SPME of PAHs. Their results showed that TiO_2_ nanotube arrays are capable of extracting PAHs, but the very thin nanotube walls make the coating very fragile and easy to destroy when carrying out SPME [27]. The Du group presented a simple and rapid anodic method for the in-situ fabrication of a novel fiber consisting of Ti wire coated with rod-like TiO_2_. It was used for the concentration and determination of trace PAHs and phthalates (PAEs) by SPME, coupled to HPLC with UV detection [28]. The Ouyang group prepared a core-shell TiO_2_@C fiber for SPME, which was carried out by the simple hydrothermal reaction of a titanium wire, followed by the coating of amorphous carbon. It was successfully used for the determination of PAHs in the Pearl River water with higher GC responses than commercial PDMS and PDMS/DVB fibers [29]. Although different metal wires have been widely used as the sorbent instead of conventional fragile silica fibers by many researchers, various sorbent coatings were always required in conjunction with the metal wire for SPME in previous reports. The Yan group fabricated a stainless steel wire etched with hydrofluoric acid for SPME [30]. Although the pristine wire had almost no extraction capability toward the tested analytes, the etched wire did exhibit a high affinity to the tested PAHs, with a high enhancement factor in the range of 2541–3981, but no extraction ability to hydrophilic phenol, butanol, and aniline was found. It was suggested that a porous and flower-like structure with Fe_2_O_3_, FeF_3_, Cr_2_O_3_, and CrF_2_ on the surface of the stainless steel wire gave a high affinity to the hydrophobic PAHs due to cation-π interaction [31]. In this case, how to evaluate the hydrophobic interaction effectively between the sorbent and target compounds in real samples is still a big challenge.

In this work, the titanium wires and meshes with different surface wettability were fabricated by a simple hydrothermal digestion and chemical immersion method. The superhydrophilic substrates with flower-like TiO_2_ nanoparticles were obtained after being etched by HF and changed to superhydrophobic after being modified using a low-surface-energy material. The micro/nano hierarchical structures and photosensitivity of the formed TiO_2_ nanoparticles on the surfaces of the Ti substrates exhibited the switchable wettability. After UV irradiation, the superhydrophobic samples became superhydrophilic. The hydrophilic, superhydrophilic, superhydrophobic, and UV-irradiated superhydrophilic wires were initially selected as the sorbents for online in-tube SPME. The morphologies and element composition of the Ti wires were observed by SEM, EDS, and XPS, and their surface wettability was measured using the Ti mesh by a contact angle goniometer. The extraction tube filled with the prepared Ti wire was connected to the injection valve of HPLC. Six common estrogenic hormones were selected as the target analytes to investigate the extraction efficiency. The online analytical method was established and used for the determination of six estrogens in water samples using the optimal conditions. More importantly, the hydrophobicity interactions between the wire surfaces and the samples were further investigated by the selection of sorbents with different surface wettabilities and the target compounds of hydrophobic PAHs.

## 2. Results and Discussion

### 2.1. Characterization

**Figure 1:**
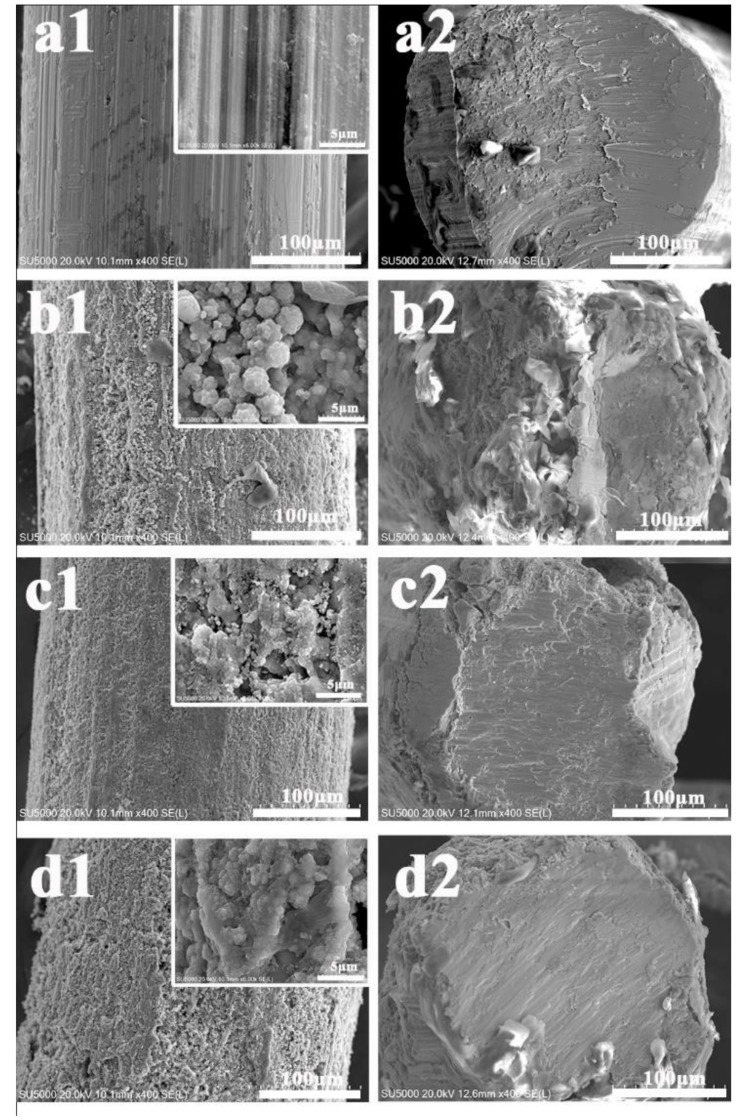
SEM for the pristine wire (**a1**,**a2**), the superhydrophilic wire (**b1**,**b2**), the superhydrophobic wire (**c1**,**c2**), and the UV-irradiated superhydrophilic wire (**d1**,**d2**).

The morphological properties and surface elemental compositions of four kinds of wires were characterized by SEM, EDS, and XRD. As can be seen in Figure 1(a1), the pristine wire had a smooth surface with few grooves. After hydrothermal digestion in strong acids at a high temperature, some small holes appeared, the surface was scattered with flower-like TiO_2_ nanostructures in Figure 1(b1), and the increase in roughness led to an increase in adsorption sites in the extraction. Further chemical modification with a low surface energy caused an observed decrease in surface wettability, which formed a superhydrophobic surface in Figure 1(c1). After UV irradiation for Ti wire c, the superhydrophobic surface transformed to superhydrophilic in Figure 1(d1). As seen in the cross-section of the wires in Figure 1(a2), no coating was observed for the smooth pristine wire. For the modified wires, the thickness of the coating was thin due to the formation of multiple layers of nanoflowers on the surface in Figure 1(b2–d2). There were some internal and external gaps in the nanoflower clusters. This coating morphology probably increases its specific surface area, improving the mass transfer and extraction efficiency.

**Figure 2:**
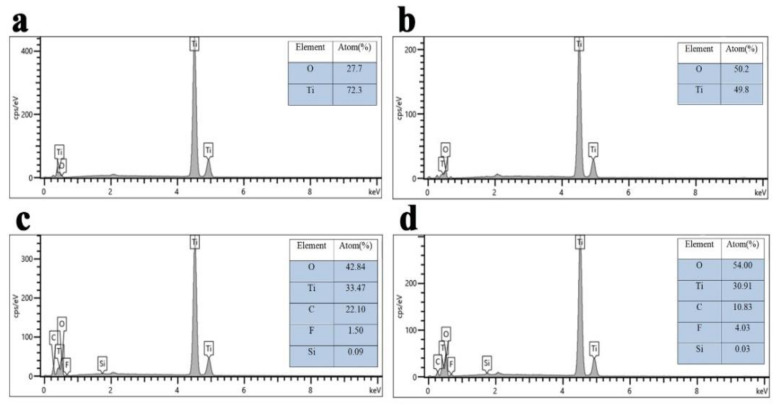
EDX for the pristine wire (**a**), the superhydrophilic wire (**b**), the superhydrophobic wire (**c**) and the UV-irradiated superhydrophilic wire (**d**).

EDS further proved the elemental changes for four kinds of Ti wires in Figure 2. The pristine Ti wire was mainly composed of Ti and O elements (a). After chemical etching, the oxygen element content increased from 27.7 to 50.2% (b). Carbon, silicon, and fluorine elements were observed after further modification of FOTS, which demonstrated thin organic films surrounding the Ti wire surface (c). Further UV irradiation led to the formation of hydrophilic domains on the TiO_2_ surface. When photosensitive TiO_2_ on the wire surface is irradiated by UV light, pairs of electron holes appear on its surface, which can react with lattice oxygen to form surface oxygen vacancies. More monolayers and multilayers of water molecules can be kinetically attached to these vacancies by molecular adsorption. It was proven by the increase in oxygen element from 42.8 to 50.0% (d).

**Figure 3:**
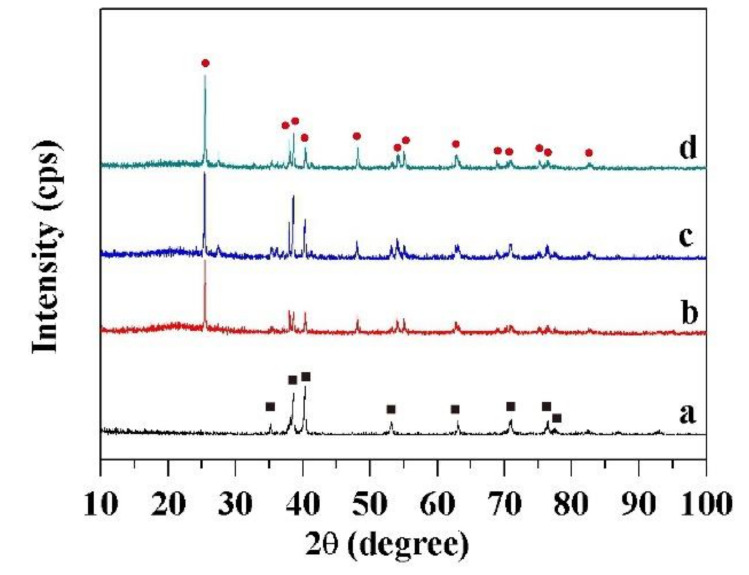
XRD patterns of Ti substrates. Symbol identification: (**a**) the pristine mesh, (**b**) the superhydrophilic mesh, (**c**) the superhydrophobic mesh, and (**d**) the UV-irradiated superhydrophilic mesh.

XRD was used to investigate the crystal structure and phase of four kinds of Ti meshes. Figure 3 presents the XRD patterns of Ti substrates with different surface wettabilities. The samples were in the anatase phase, and the diffraction peaks were indexed to that of anatase (JCPDS No. 21-1272) [32]. No characteristic peaks from impurities were detected within experimental error, indicating the formation of anatase nanostructures.

In the 2θ scan range 10°–100°, all show characteristic peaks of Ti(100), Ti(002), Ti(101), Ti(102), Ti(110), Ti(103), Ti(112), and Ti(201) at 35.1°, 38.4°, 40.2°, 53.0°, 62.9°, 70.6°, 76.2°, and 77.3°, respectively. These data for the pristine (a) are in good agreement with the Ti crystallographic data (Reference code:00-001-1198). In comparison, all show characteristic peaks of TiO_2_(101), TiO_2_(103), TiO_2_(004), TiO_2_(112), TiO_2_(200), TiO_2_(105), TiO_2_(211), TiO_2_(204), TiO_2_(116), TiO_2_(220), TiO_2_(107), TiO_2_(301), and TiO_2_(312) at 25.3°, 36.9°, 37.8°, 38.6°,48.0°, 53.9°, 55.1°, 62.7°, 68.8°, 70.3°, 74.1°, 76.0°, and 83.1°. The 13 peaks are due to the formation of Ti oxides in the crystalline phase, which are in accordance with the TiO_2_ crystallographic data (Reference code:03-065-5714). Although the presence of crystal structures is not a differentiated factor for the hydrophobicity or hydrophilicity of the surfaces, a significant difference was obviously found between the pristine Ti surface (a) and other three Ti surfaces with nano TiO_2_ particles in Figure 3b–d.

### 2.2. Evaluation of Surface Wettability for the Prepared Wires

**Figure 4:**
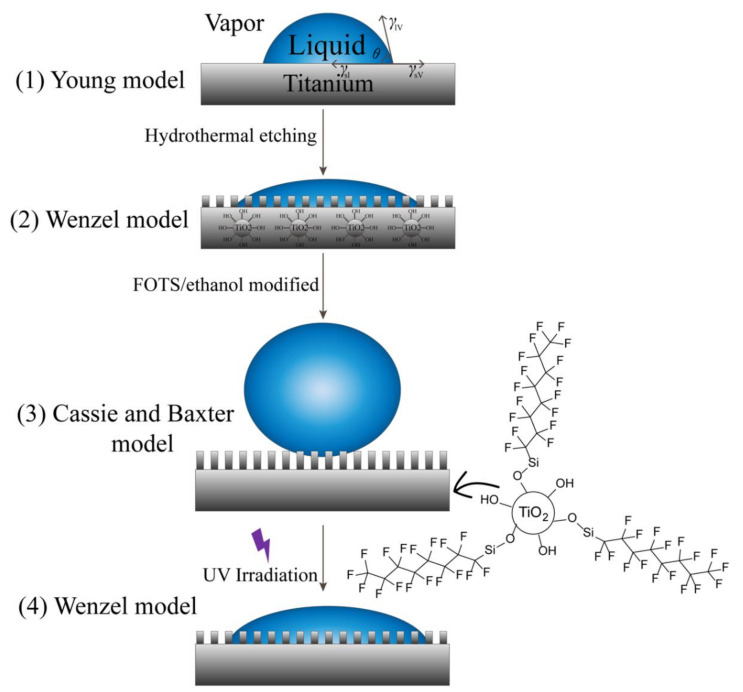
Model representation of the surface wettability for the preparation of Ti wires and meshes. The resulting Ti meshes with 3 typical surface states: (**1**) Young Model, (**2**,**4**) Wenzel Model, and (**3**) Cassie and Baxter Model.

The preparation of superhydrophobic surfaces on hydrophilic metal substrates requires surface micro/nano-structures and low surface energy modification. As shown in Figure 4, there are three typical models to illustrate the surface states, which were developed by Young [33], Wenzel [34], and Cassie and Baxter [35].

Before the hydrothermal process, the surface of the Ti substrate is relatively smooth with a hydrophilic property, which is in accordance with Model 1. After hydrothermal treatment by HF about 8 h, the Ti substrate was chemically etched, and the surface became rougher with more porous structures. A few of nanoparticles were also found on the surface with superhydrophilicity, which is close to Model 2. The readily hydrothermal procedure afforded the in-situ synthesis of TiO_2_ nanowires on the Ti substates and provided a desirable support for the further modification of a low-surface-energy material. TiO_2_ nanoparticles are hydrophilic, which results from the large number of hydroxyl groups on their surface. After the Ti substrate was self-assembled by FOTS, the surface energy was apparently reduced [36]. In this case, the silicon ethoxide groups (Si–Cl) in FOTS were hydrolyzed to silanol groups (Si–OH). FOTS, as a low-surface-energy material then reacted with the –OH groups of the TiO_2_ surface. The superhydrophobic coating by the Si–O–Ti bond is in accordance with Mode 3. The superhydrophobic surface was changed to superhydrophilic after UV irradiation, which was in the state of Mode 1.

**Figure 5:**
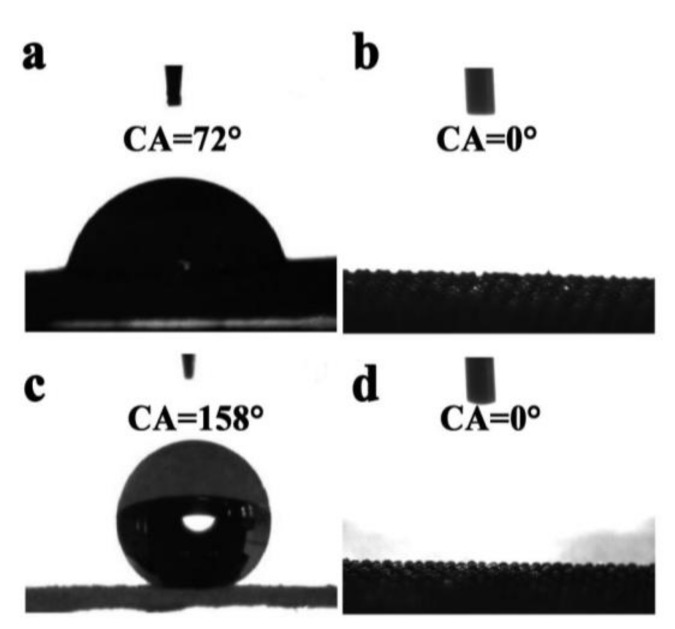
WCAs of the pristine Ti mesh (**a**), the etched Ti mesh (**b**), the FOTS-modified Ti mesh (**c**) and the UV-irradiated superhydrophilic mesh (**d**).

The Ti meshes that were prepared using an identical method were used for the comparative determination of surface wettability instead of Ti wires. The pristine mesh displayed a static water contact angle of about 72° (Figure 5a). After it was etched by 20 mM HF for 6 h by hydrothermal digestion, the water droplets almost spread on the mesh completely (Figure 5b). When the etched mesh was immersed in FOTS for 1 h, the surface became superhydrophobic, with a WCA of 158° (Figure 5c). After UV irradiation for about 15.5 h, it changed to superhydrophilic, with a CA of nearly zero degrees (Figure 5d). A large group of hydrophilic and oleophilic domains was thus created on the surface of the Ti substrate. Moreover, the surface wettability of the Ti substrate could be reversibly switched between superhydrophilicity and hydrophobicity under the alternation of UV light illumination and long-term dark storage, independent of their photocatalytic activities [37,38].

### 2.3. Extraction Efficiency for the Polar Compounds

**Figure 6:**
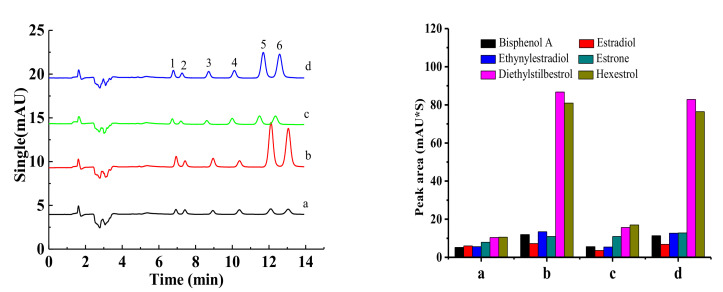
Chromatograms of the target compounds using four kinds of fibers with different surface wettabilities (**Left**) and the comparative peak area for each target compound using 4 kinds of wires for in-tube SPME (**Right**). Symbol identification: (a) pristine wire, (b) superhydrophilic wire, (c) superhydrophobic wire, (d) superhydrophilic wire after UV irradiation. Conditions: sampling volume, 60 mL; sampling rate, 2.00 mL/min; desorption time, 2.0 min. Peak identification: (1) bisphenol A, (2) estradiol, (3) ethynylestradiol, (4) estrone, (5) diethylstilbestrol, and (6) hexestrol. Mobile phase: Water/acetonitrile (53:47, *v/v*); Detection wavelength, 220 nm, room temperature.

Four kinds of Ti wires were inserted into the extraction tube for the comparative investigation of extraction efficiency. Benefiting from the much larger surface area of the subsequent TiO_2_ around their surface and different surface energy after hydrothermal etching and chemical modification, the superhydrophilic wire (b) and UV-irradiated superhydrophilic wire (d) exhibited higher extraction efficiency than the pristine and superhydrophobic wires in Figure 6 (left). Two kinds of superhydrophilic Ti wires showed a small enrichment for the first four compounds, including bisphenol A, estradiol, ethynylestradiol, and estrone, and a particularly large enrichment for the last two compounds, including diethylstilbestrol and hexestrol. The comparative peak areas for each target compound are illustrated in Figure 6 (right). Superhydrophilic materials, due to their intrinsic character of polar nature, are expected to be the perfect option for the extraction of organic species with more polarity.

The enrichment factor (EF) values were accurately calculated as follows [3,4,7]: After 20 μL of standard solutions with different concentrations of 0, 0.25, 0.5, 1, 2.5, 5, 10, 25, and 100 mg/L were injected using the sample loop, the calibration curve with a slope of k_1_ was then calculated according to the peak area vs. concentration. Similarly, another series of standard solutions with a constant sampling volume of 60 mL and different concentrations of 0, 0.5, 1, 2, 5, and 10 μg/L were prepared. After they were pumped into the extraction tube for preconcentration, each analyte in the standard solution was desorbed and detected through the UV detector. The calibration curve was then achieved with a slope of k_2_. The final EF can be calculated according to the following equation: EF = k_2_/k_1_.

### 2.4. Optimization of Extraction Conditions

**Figure 7:**
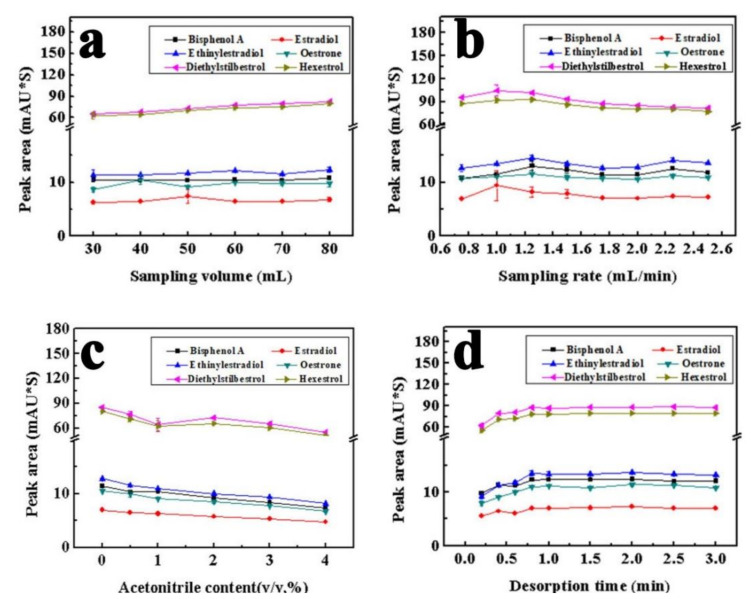
The optimization of extraction conditions using the superhydrophilic fiber as the sorbent for in-tube SPME coupled with HPLC. The concentration of each estrogen is 10 μg/L. Other experimental conditions are the same as Figure 6. Symbols of (**a**–**d**) stand for the relationship between the change of peak area with the sampling volume, sampling rate, ACN content and desorption time.

To achieve the highest sensitivity, some important factors affecting the extraction efficiency were studied, including the sampling volume, sampling rate, acetonitrile content in the sample, and desorption time [39]. When the peak area tends to be constant with the increase in the sample volume, the extraction equilibrium and the largest extraction efficiency will be obtained. However, an excessive sampling volume consumes more extraction and desorption time. As shown in Figure 7a, the peak areas of the analytes, especially for hexestrol and diethylstilbestrol, increased with the change of the sampling volume from 30 to 80 mL. The increase in the peak areas was unremarkable for other compounds when the sampling volume was more than 60 mL. Compromising the extraction efficiency and time, 60 mL was selected as the optimal sampling volume.

After the same sampling volume of 60 mL was loaded, the sampling rate was investigated in the range of 0.75–2.50 mL/min. It can be seen in Figure 7b that the peak areas of the six analytes changed little when increasing the sampling rate from 0.75 to 2.50 mL/min. While the sampling rate increases beyond 1.00 mL/min, the downward trend occurs for hexestrol and diethylstilbestrol. In order to obtain better extraction efficiency and quick analysis for the six analytes, 2.00 mL/min was selected as the optimal sampling rate for overall consideration.

Organic solvents can promote the solubility of hydrophobic analytes in an aqueous sample and improve analysis repeatability. Herein, acetonitrile was selected as the organic solvent to investigate the effect of organic compounds in the sample on the extraction process. The content of acetonitrile was varied from 0 to 4% (*v/v*), and the peak areas of the analytes were investigated. As shown in Figure 7c, except for hexestrol and diethylstilbestrol, the extraction efficiency of the other four analytes decreased with the addition of acetonitrile in the sample. The peak area of diethylstilbestrol has a little increasing trend from 1 to 2% and then a decreasing trend from 2 to 4%. In order to obtain satisfactory extraction efficiency, no acetonitrile was added to the samples.

A full desorption of all extracted analytes can be obtained after adequate desorption time, which thus reduces the impact of residual analytes on the next extraction. After the extraction was completed, acetonitrile–water (53:47) as the mobile phase flowed through the extraction tube at 1.00 mL/min. The analytes absorbed by the sorbent in the extraction tube were eluted and the desorption process was carried out. The effect of desorption time was varied in the range of 0.20–3.00 min. As shown in Figure 7d, the six estrogen peak areas exhibited an upward trend with the increase of the desorption time. Therefore, 2.00 min was selected as the optimal desorption time.

**Figure 8:**
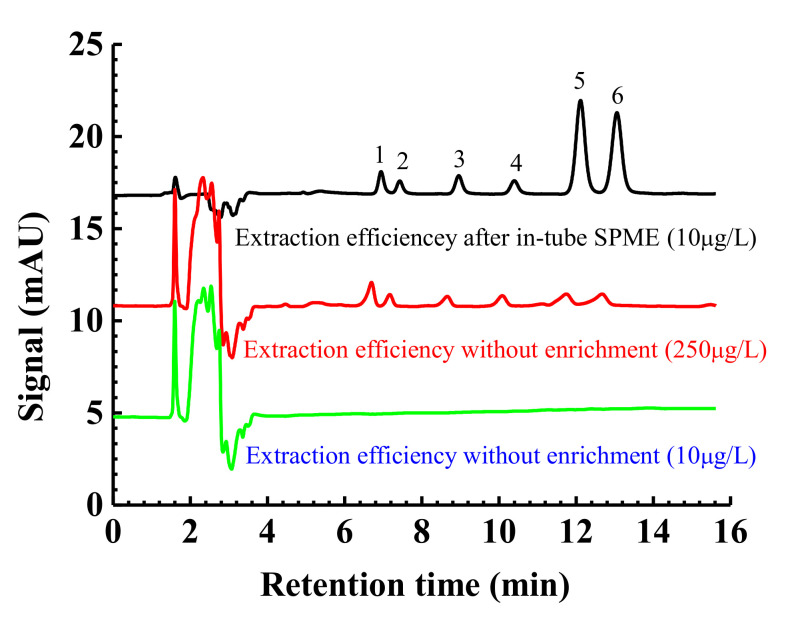
Comparative HPLC chromatograms before and after enrichment using the superhydrophilic wire or in-tube SPME. Peak order and other experimental conditions are the same as Figure 6.

Based on the above discussion, the optimal conditions of in tube SPME for estrogens are: 60 mL sampling volume, 2 mL sampling rate, 2 min desorption time at a flow rate of 1 mL/min. Under the optimized extraction conditions, the expected extraction performance of superhydrophilic wire (b) for the target analytes was achieved. As shown in Figure 8, the estrogen analytes were not detected for the direct injection of a sample spiked at 10 μg/L. After the concentration of each estrogen compound increased to 250 μg/L, some small peaks of the six estrogens appeared. Compared with the signals after the extraction by the proposed in-tube SPME method, chromatographic peaks become very obvious, especially for diethylstilbestrol and hexestrol. A higher extraction efficiency indicated that there were abundant multiple interactions between the superhydrophilic wire (b) and the target analytes.

### 2.5. Method Evaluation and Application to Real Samples

Under the optimal conditions, a series of parameters were investigated to validate the online in-tube-SPME-HPLC method, which included linear range, correlation coefficient (r), inter-day repeatability, intra-day repeatability, limits of detection (LODs), and limits of quantification (LOQs). As shown in Table 1, the analysis method was established with the linear range of 0.5–10.0 μg/L, the correlation coefficient (R^2^) of 0.9794–0.9910, and the LOD of 0.08–0.14 μg/L for the six estrogens. The relative standard deviations (*n* = 3) for the intra-day (≤2.1%) and inter-day (≤4.8%) tests demonstrated that satisfactory repeatability was obtained for the superhydrophilic wire. Each estrogen component on the extraction tube was enriched effectively with their EF values in the range of 20–177.

To confirm the applicability of the analytical method, tap water taken from our laboratory were selected for the analysis. As shown in Table 2, none of analytes were detected in the tap water. The spike recoveries of the analytes were determined by the standard addition method. The concentration of addition in the sample was 0, 5, and 10 μg/L. The recoveries of the six analytes were in the range of 73.2–91.1%, indicating that this method could be applied to analyze trace estrogens in real samples.

### 2.6. Extraction Efficiency for Non-Polar Target Compounds

**Figure 9:**
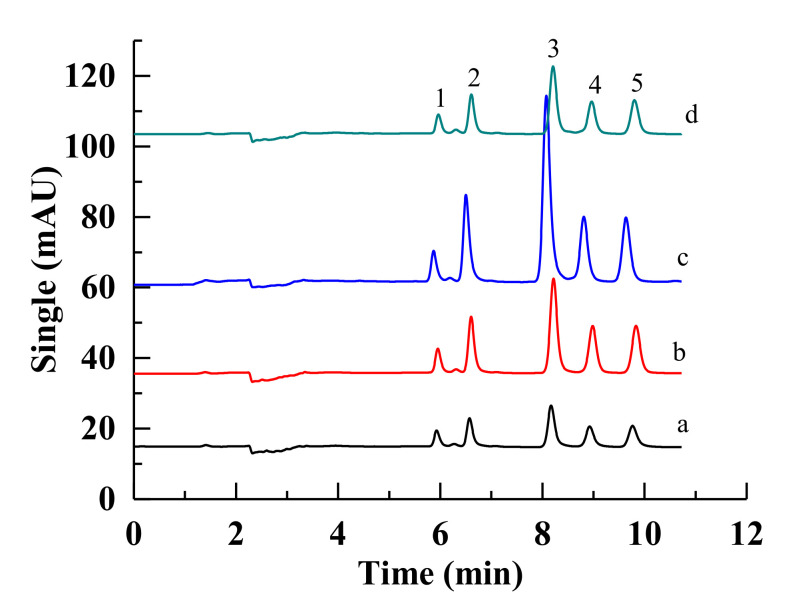
Chromatograms of the target compounds using four kinds of fibers (a, b, c, d) with different surface wettability. The concentration of each compound of PAHs is 10 μg/L. Peak identification: 1. naphthalene, 2. acenaphthylene, 3. acenaphthene, 4. phenanthrene, 5. Anthracene. Water/acetonitrile (25:75, *v/v*), Detection wavelength, 226 nm. Other conditions are the same as Figure 6.

The correlation of the surface wettability for the sorbent and the hydrophobicity of the target compounds in the sample matrix was further investigated. Superhydrophobic materials were expected to be the ideal sorbent for the enrichment of organic species with the least amount of polarity [40,41,42]. Herein, the lowest peak areas for five PAHs were obtained using the pristine hydrophilic wire (a) in Figure 9. In comparison, both superhydrophilic wires (b,d) exhibited similar enrichment abilities. It should be noted that the superhydrophobic wire (c) had the highest extraction efficiency to the non-polar compounds of five PAHs. It demonstrated that the surface wettability of the sorbent resulted in different effects for the preconcentration of target compounds. Most artificial superhydrophobic surfaces usually present a rough structure in the micro- or nanoscale and have poor mechanical and chemical stability. Herein, the fabricated superhydrophobic Ti wires can endure a long-time rinse from the mobile phase without a decrease in hydrophobicity and enrichment ability. In order to confirm the truth further, a superhydrophobic Ti mesh was immersed in the mobile phase solution for one week and sonicated discontinuously. The highly hydrophobic property still remained for the dried mesh. It indicated our fabricated superhydrophobic wire with strong mechanic and chemical stability could be used as a robust sorbent for in-tube SPME.

In general, the enrichment capability is positively related to the absorption or adsorption capability of the coating material [17]. Herein, we first systematically elucidated the relationship between the surface wettability of the sorbent and the polarity of the target analytes by selecting a smart material with switchable surface wettability. It is similar to the theory of “like dissolves like”, primarily determining the selectivity of a self-made coating by the hydrophobic/hydrophilic interaction.

## 3. Materials and Methods

### 3.1. Materials and Reagents

Ti wire (purity 99.9%) with a diameter of 0.3 mm, Ti mesh (3 cm wide × 6 cm long × 0.8 mm thick) and stainless tube (0.8 mm I.D, 1.6 mm O.D) were purchased from Shenzhen Global Copper and Aluminum Materials Co., Ltd., Shenzhen, China. 1*H*,1*H*,2*H*,2*H*-Perfluorooctyltrichlorosilane (97%) was purchased from Shanghai Macklin Biochemical Technology Co., Ltd., Shanghai, China. Methanol and Acetonitrile were HPLC grade and were purchased from Tianjin Damao Reagent Company (Tianjin, China). Polar estrogens, including bisphenol A (>99.0%), ethinylestradiol (>98.0%), estradiol (>98%), diethylstilbestrol (>99.0%), hexestrol (>98.0%), and estrone (>98.0%), and non-polar PAHs, including naphthalene, acenaphthylene, acenaphthene, phenanthrene, and anthracene, were purchased from Shanghai Aladdin Biochemical Co., Ltd. (Shanghai, China). Ultrapure water (18.25 MΩ cm, 25 °C) was used for the whole experiment. Water/acetonitrile (53:47, *v/v*) was used as the mobile phase to elute estrogens at the detection wavelength of 202 nm.

### 3.2. Apparatus

An EasySep-1020LC pump (Elite instrumental, Dalian, China) was used to transport sample solutions. Analytes were detected by the Agilent 1220 HPLC system equipped with a Hypersil ODS-C18 column (250 × 4.6 mm i.d., 5 μm, Elite instrumental) and a variable wavelength detector (VWD). The prepared wires were characterized by an SEM (quanta 200 environmental scanning electron microscope) and X-ray photoelectron spectra (XPS, Thermo ESCALAB 250XI, Thermo Fisher Scientific, Bohemia, NY, USA). The contact angles were measured by the pendent drop method using a CA goniometer (TST-200, Shen Zhen Testing Equipment Co., Ltd., Shen Zhen, China). The materials were UV-irradiated by an instrument of UV crosslinker (SCIENT03-II, Xin-zhi Biotechnology Co., Ltd., Lingbo, China). Four UV tubes (254 nm) were installed on the top of the UV oven, and the distance between the irradiated samples on the bottom of the UV oven and the upper tube was about 11 cm.

### 3.3. Standard Solution and Real Samples

The stock solution (100 mg/L) containing five estrogens was prepared with methanol and stored at 4 °C. The working solution was prepared daily by dilution of the stock solution with pure water to 10 μg/L. Tap water taken from our laboratory was selected as the real samples for the evaluation. All water samples were filtered with a 0.45 μm water membrane of cellulose ester before chromatographic analysis.

### 3.4. Preparation of Ti Wires with Different Surface Wettabilities

The pristine Ti wires and Ti meshes were ultrasonically cleaned by acetone and water consequentially for 5 min and then dried. The cleaned Ti wires (30 cm long, 0.3 mm I.D) and Ti meshes were immersed in 18% HCl solution at 85 °C for 15 min. After that, these samples were placed in a PTFE-lined stainless steel autoclave containing 20 mM hydrofluoric acid. The autoclave was sealed, and the Ti samples were etched under hydrothermal conditions at 160 °C for 8 h [43,44].

In this hydrothermal process, HF not only etches the Ti substrate, providing a Ti source for the formation of TiO_2_ nanostructures but also serves as the source of F-dopant. After hydrothermal synthesis, the samples were rinsed thoroughly with deionized water and dried with nitrogen. Then, the superhydrophilic Ti samples were obtained.

The resulting superhydrophilic samples were immersed in 1% ethanol solution of heptadecafluoro-1,1,2,2-tetrahydrodecyl trichlorosilane (FOTS) for 1 h. Superhydrophobic surfaces with flower-like TiO_2_ nanostructures were then obtained for the Ti wires and meshes. The superhydrophobic surfaces were changed to superhydrophilic after UV irradiation for about 15.5 h using a UV crosslinker.

**Figure 10:**
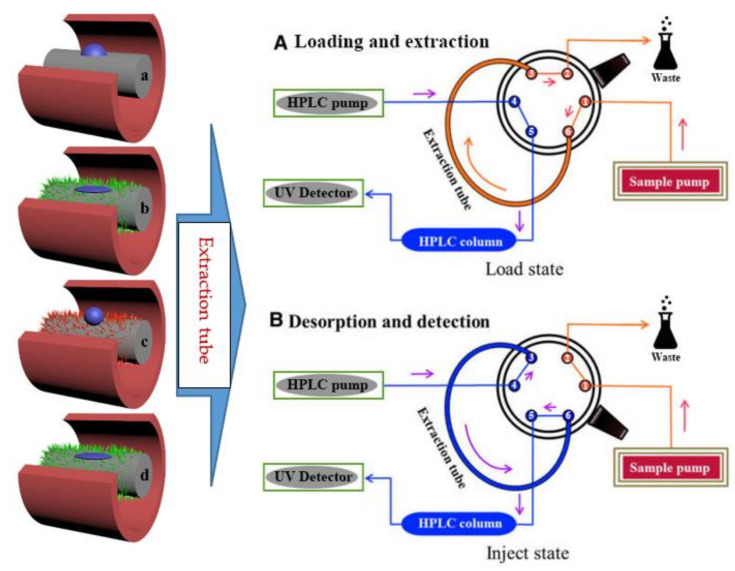
(**Left**) The fabricated wire with different surface wettability was inserted into the microextraction tube. Symbol identification: (a) the pristine wire, (b) the superhydrophilic wrie, (c) the superhydrophobic wire and, (d) the UV-irradiated superhydrophilic wire. (**Right**) Schematic illustration of in-tube SPME–HPLC online system.

Afterwards, as shown in Figure 10(left), the pristine wire (a), the superhydrophilic wire (b), the superhydrophobic wire (c), and the UV-irradiated superhydrophilic wire (d) with a length of 30 cm were filled into stainless-steel tubes (0.75 mm i.d. and 1/16 inch o.d.).

### 3.5. Extraction and Analysis Procedure

As shown in Figure 10(left), the extraction tube was connected to the six-port valve instead of the sample loop. The six-port high pressure valve was switched between the load and inject states to perform the extraction and desorption processes. In the load state, the sample solution was transported through the extraction tube via an EasySep-1020LC pump, and the target analytes were adsorbed by the selected wire. The sample was pumped and flushed from pore 1 to pore 6, then pressured into the extraction tube and entered pore 3. After the preconcentrated target compounds in sample were continually entrapped by the extraction materials, the remaining solution was flushed to the waste bottle from pore 2. After the rotor was rotated about 60°, the system changed to the injection state. In this case, the mobile phase flowed through the extraction tube from pore 4 to pore 3, the absorbed target compounds were desorbed by the mobile phase, and the mobile phase with the desorbed compounds was pumped to the separation column from pore 6 to pore 5 for UV detection, consequently. The extraction and the desorption could be completed by the switch between the load and inject modes in Figure 10(right).

## 4. Conclusions

In summary, flower-like nano TiO_2_ surfaces with switchable wettability were successfully used to investigate the hydrophobic interaction for in-tube SPME. The prepared Ti wires with different surface wettabilities were first compared as a novel extraction support for an online in-tube SPME-HPLC. The results showed that the superhydrophilic wire (b) and the UV-irradiated superhydrophilic wire (b) exhibited the highest extraction efficiency for polar compounds of estrogens compared with the pristine hydrophilic wire (a) and superhydrophobic wire (c). In contrast, the superhydrophobic wire (b) had the higher extraction efficiency for non-polar PAHs compared with the other three wires. We firstly demonstrated that the surface wettability of sorbent might affect the enrichment of target compounds with different polarity effectively. How to understand the theory of like dissolves like from the surface wettability of the sorbent should be further investigated in our future work.

## Figures and Tables

**Table 1:** Analytical performances and EF values of six estrogens using in-tube SPME-HPLC method.

Analytes	LODs (μg/L)	LOQs (μg/L)	Linear Ranges (μg/L)	R^2^	EF	Extraction Repeatability (*n* = 3, RSD%) ^a^		Preparation Repeatability (*n* = 3, RSD%) ^b^
						Intraday	Interday	
Bisphenol A	0.097	0.323	0.5–10.0	0.9857	20	2.0	4.6	10.4
Estradiol	0.135	0.450	0.5–10.0	0.9886	33	2.1	4.8	8.9
Ethynyl estradiol	0.131	0.437	0.5–10.0	0.9886	28	1.3	3.2	5.2
Estrone	0.079	0.263	0.5–10.0	0.9867	177	0.8	4.5	14.3
Diethylstilbestrol	0.132	0.440	0.5–10.0	0.9794	43	1.7	4.0	2.3
Hexestrol	0.092	0.307	0.5–10.0	0.9910	154	0.9	2.9	9.4

^a^ Extraction repeatability was investigated by extracting 10 μg/L of estrogens three times. ^b^ Preparation repeatability was investigated by extracting 10 μg/L of estrogens with three extraction tubes.

**Table 2:** Analysis results and recoveries (%) of six estrogens in tap water.

Analytes	Bisphenol A	Estradiol	Ethynyl Estradiol	Estrone	Diethylstilbestrol	Hexestrol
Recovery (5 μg/L, %)	76.2 5.0	89.4 0.2	86.6 1.6	78.4	91.1 1.5	82.7 2.4
Recovery (10 μg/L, %)	87.7 5.3	76.4 0.6	74.6 0.8	75.3	73.2 4.4	87.4 2.4
Tap water (μg/L)	Not detected	Not detected	Not detected	Not detected	Not detected	Not detected

## Data Availability

Not applicable.

## Sample Availability

Samples of the compounds are available from the authors.
